# Longitudinal investigation of gait and Alzheimer's disease in adults with Down syndrome

**DOI:** 10.1002/alz.70211

**Published:** 2025-04-28

**Authors:** Ashlyn Barry, Jamie C. Peven, Benjamin L. Handen, Daniel Bolt, Sharon J. Krinsky‐McHale, Christy L. Hom, Isabel C. H. Clare, Amanda Glueck, Jordan Harp, Frederick Schmitt, Matthew Zammit, Davneet Minhas, Weiquan Luo, Charles Laymon, Julie Price, Joseph H. Lee, Ira Lott, Annie Cohen, Beau M. Ances, Margaret Pulsifer, H. Diana Rosa, Florencia Lai, Shahid H. Zaman, Elizabeth Head, Mark Mapstone, Bradley T. Christian, Sigan L. Hartley, the Alzheimer Biomarker Consortium ‐ Down syndrome

**Affiliations:** ^1^ Waisman Center University of Wisconsin–Madison Madison Wisconsin USA; ^2^ Behavioral Health Service Line VA Pittsburgh Healthcare System Pittsburgh Pennsylvania USA; ^3^ Department of Psychiatry University of Pittsburgh Pittsburgh Pennsylvania USA; ^4^ New York Institute for Basic Research in Developmental Disabilities Department of Psychology Staten Island New York USA; ^5^ Deparment of Psychiatry & Human Behavior University of California, Irvine Irvine California USA; ^6^ Department of Psychiatry University of Cambridge Cambridge UK; ^7^ Department of Neurology University of Kentucky Lexington Kentucky USA; ^8^ Department of Radiology University of Pittsburgh Pittsburgh Pennsylvania USA; ^9^ Department of Bioengineering University of Pittsburgh Pittsburgh Pennsylvania USA; ^10^ Department of Neurology Massachusetts General Hospital Harvard Medical School Boston Massachusetts USA; ^11^ Taub Institute for Research on Alzheimer's Disease and the Aging Brain Sergievsky Center and Department of Neurology Vagelos College of Physicians and Surgeons Columbia University New York New York USA; ^12^ Department of Pediatrics University of California Irvine School of Medicine Irvine California USA; ^13^ Department of Neurology Washington University School of Medicine in St. Louis St. Louis Missouri USA; ^14^ Department of Pathology & Laboratory Medicine University of California, Irvine School of Medicine Irvine California USA; ^15^ Department of Neurology University of California Irvine School of Medicine Irvine California USA; ^16^ Department of Human Development and Family Studies University of Wisconsin–Madison Madison Wisconsin USA

**Keywords:** amyloid beta, cognitive decline, dementia, gross motor skills, hippocampal volume, neurofibrillary tangles of tau, trisomy 21

## Abstract

**INTRODUCTION:**

Gait abnormalities are associated with Alzheimer's disease (AD) in the general population, but it is unclear if the same is true for individuals with Down syndrome (DS). This study examined gait across 32 months in relation to neuroimaging biomarkers (amyloid beta [Aβ], neurofibrillary tangles [NFTs], and hippocampal volume), cognitive decline, and clinical AD status in adults with DS.

**METHODS:**

Participants were 218 adults with DS who underwent Aβ and NFT positron emission tomography (PET) and magnetic resonance imaging (MRI) scans, cognitive testing, and gait assessments at baseline and 32 months. Residual change regression models were conducted.

**RESULTS:**

Higher baseline Aβ PET and NFT PET and lower MRI hippocampal volume were associated with gait declines across 32 months. Cognitive declines were associated with gait declines. Participants with clinical dementia at 32 months had greater gait decline than those who were cognitively stable.

**DISCUSSION:**

Gait impairments are a key feature of DS‐associated AD (DSAD). Gait assessments could offer a quick, cost‐effective, non‐invasive screen for DSAD.

**Highlights:**

- Those with clinical status of dementia had lower gait performance than those who were cognitively stable.
- Higher baseline amyloid beta and neurofibrillary tangle volume was associated with more gait impairments.
- Lower baseline hippocampal volume was associated with more gait impairments.
- Greater decline in gait performance was associated with cognitive decline.
- Greater decline in gait performance was associated with more dementia symptoms.

## 1 BACKGROUND

Down syndrome (DS) is a developmental disability caused by an extra copy of chromosome 21^1^ that occurs in ≈ 1 in 700 live births in the United States.^2^ Adults with DS have a 90% lifetime risk for Alzheimer's disease (AD) dementia,^3^ which is thought to be driven by the triplication of the amyloid precursor protein (APP) gene located on chromosome 21.^4^ The presence of AD pathology in DS is similar to sporadic AD; however, the accumulation of amyloid beta (Aβ) brain plaques occurs several decades earlier,^5^, ^6^, ^7^, ^8^ followed by the presence of neurofibrillary tau tangles (NFTs),^9^, ^10^, ^11^, ^12^ and then neurodegeneration as evidenced by hippocampal atrophy, altered glucose metabolism, and increased glial fibrillary acidic protein levels.^13^, ^14^ Over the past decade, the timeline of cognitive changes that reflect mild cognitive impairment (MCI) and dementia in DS‐associated AD (DSAD) has been described^15^, ^16^, ^17^, ^18^, ^19^ and more recently the relationship with Aβ plaques and NFTs.^20^ However, little is known about other domains of changes in daily functioning during the progression to DSAD.

In the general adult population, changes in gait have long been associated with AD.^21^, ^22^, ^23^ Changes in gait were initially found to be associated with later stages of AD, after the onset of dementia.^24^, ^25^ Indeed, there is substantial evidence that adults with AD dementia experience impairments in balance and gait,^26^, ^27^, ^28^ such as increased postural sway^29^ and reduced mobility.^30^ Recently, gait impairments have also been observed in individuals with MCI.^31^, ^32^, ^33^ Additionally, gait impairments appear to be associated with the earliest stages of AD development, such as early‐stage Aβ deposition.^32^ The etiology of these gait impairments may be related to disruption of distributed thalamo–cortico–basal ganglia networks related to motor control^33^, ^34^ or to more focal changes of frontal cognitive–motor cortical regions.^35^, ^36^

It remains uncertain whether changes in gait are a feature of DSAD. Individuals with DS have lifelong difficulties with gait,^37^ and additional gait impairments may occur as early as the mid‐30s.^38^ In part, changes in gait in middle and late adulthood may be driven by the elevated prevalence of aging‐related conditions in DS, such as sarcopenia and osteoporosis,^39^, ^40^, ^41^ as well as atlantoaxial subluxation throughout the lifespan.^39^ However, AD pathology may also contribute to gait impairments.^42^ In a dual‐task assessment (counting while walking), Van Pelt et al.^43^ found that adults with DS who had AD dementia showed significant reductions in gait velocity compared to those who were cognitively stable. However, this finding may be due to the cognitive load required for a dual‐task assessment. In a different study (*n* = 66), the Tinetti Performance Oriented Mobility Assessment^44^ (POMA, also known as Tinetti) Gait subscale distinguished adults with DS with MCI and dementia from those who were cognitively stable.^45^ The current study built on these prior studies by evaluating change in gait across 32 months in relation to positron emission tomography (PET) and magnetic resonance imaging (MRI) biomarkers of AD pathology, cognitive decline, and clinical AD status in a sample of 218 adults with DS. The study aims were to: (1) examine gait across 32 months in relation to baseline biomarkers of Aβ PET, NFT PET, and MRI hippocampal volume; (2) determine whether change in gait across 32 months is associated with change in cognitive functioning during this same interval; and (3) compare changes in gait across 32 months based on clinical AD status (cognitively stable, MCI, or dementia). Based on prior research,^43^, ^45^ we hypothesized that changes in gait would be associated with higher baseline Aβ and NFT PET, lower MRI hippocampal volume, and greater decreases in cognitive functioning across 32 months. We also predicted that adults with DS who were classified as MCI and dementia at 32 months would exhibit greater declines in gait than those who were cognitively stable.

## 2 METHODS

### 2.1 Participants

Participants were 218 adults with DS enrolled in the Alzheimer Biomarkers Consortium‐Down Syndrome (ABC‐DS), a natural history study of DSAD that includes eight data collection sites in the United States and one in the United Kingdom. Study inclusion criteria included ≥ 25 years of age, mental age ≥ 4 years, genetic confirmation of DS (trisomy 21, mosaicism, or translocation), and availability of a study partner who could provide information on the participant with DS's medical history and daily function. Exclusion criteria at study entry included contraindications to neuroimaging (e.g., metallic implants), untreated medical conditions that could impair cognitive function, and no prior diagnosis of AD or concerns about dementia for participants who were not part of legacy studies leading up to ABC‐DS. Informed consent and/or assent were obtained from participants prior to study activities. The study was approved by institutional review boards and conducted in accordance with the Declaration of Helsinki.

### 2.2 Procedures

At each data collection cycle, the adult with DS and study partner participated in a multiday study visit. The study partner was either a parent, sibling, or caregiver who knew the participant very well. During this visit, the participant completed a neuropsychological battery designed to assess cognitive, gait, and other gross motor functions. We used three cognitive measures from this battery (see Handen et al.^46^ for full battery) shown to be valid and sensitive to AD‐related cognitive decline in DS.^47^ The study partner reported on the participant's medical history and cognitive and behavioral functioning. The participant with DS also underwent MRI and Aβ and NFT PET scans and a blood draw. For the current study, we analyzed data collected at the baseline study visit and 32‐month study visit of ABC‐DS.

**RESEARCH IN CONTEXT**

1. **Systematic review**: Theoretical and empirical literature on gait changes in Down syndrome–associated Alzheimer's disease (DSAD) and in other forms of AD was reviewed using research search databases. Relevant prior work, including cross‐sectional studies on gait in DSAD, is cited.
2. **Interpretation**: Findings indicate that gait changes are part of DSAD, occurring alongside cognitive decline, dementia status, and neuroimaging biomarkers of amyloid, tau, and hippocampal volume. Gait assessments should be part of screening for DSAD.
3. **Future directions**: Future studies using more sensitive technology‐based gait mats may be able to detect even earlier subtle changes in gait related to AD pathology and symptomology. The specific domains of gait (e.g., cadence, foot clearance, and step symmetry) impacted by AD pathology and those best able to distinguish among clinical status groups should be identified in future research.

### 2.3 Measures

#### 2.3.1 Demographic and health

The study partner reported participant demographic information. Collected information included age (in years), sex (1 = male, 2 = female), and ethnicity/race (categories: Hispanic or Latino, White non‐Hispanic, Black, Asian, Native Hawaiian or other Pacific Islander, American Indian or Alaska Native, or multiple races). The study partner also reported on the participant's medical history. In the present study, we examined the presence (vs. absence) of the 11 medical conditions for any associations with gait. These conditions were: Parkinson's disease, tremors, seizures, myoclonus, traumatic brain injury, cataracts, vision impairments, hearing impairments, osteoarthritis, osteoporosis, and gout. We determined apolipoprotein E (*APOE*) allele ε4 carrier status via genetic testing using blood cell DNA. The level of intellectual disability (ID) prior to any AD‐related condition was estimated using the Kaufman Brief Intelligence Test, Second Edition (KBIT‐2^48^) or Stanford‐Binet Fifth Edition Abbreviated Battery (SB5^49^), conducted as part of ABC‐DS prior to concerns about dementia. For the subset of participants with concerns about MCI or dementia upon entry into ABC‐DS, medical records were reviewed to find IQ scores from an earlier prior point. Our estimated level of ID was coded as (1) mild, (2) moderate, and (3) severe/profound, which corresponded to the following mental ages: mild: 9 to 14 years, moderate: 4 to 8 years, and severe/profound: ≤ 3 years. Mental age standard scores were used instead of IQ standard scores due to floor effects on the SB5 Abbreviated IQ Battery and KBIT‐2, where the lowest possible IS standard score is 40 or 46, respectively, and thus does not allow differentiation between individuals with moderate and severe/profound ID.

#### 2.3.2 Gait

The Tinetti Gait and Balance assessment^44^ was used to measure gait. Only the gait sub‐assessment was administered. Gait was assessed during two 15‐foot walks: the first walk was completed at the participant's preferred pace and the second walk was at a rapid but safe pace. Participants were scored in eight domains: indication of gait, step length and height, foot clearance, step symmetry, step continuity, path, trunk, and walking time. Each domain is scored from 0 to 1 (4 items) or 0 to 2 points (4 items), with a maximum of 12 points. Lower scores indicate greater gait impairment and higher risk of falls.^44^ The Tinetti has strong reliability and validity in the general elderly population^50^ and populations with gait impairments.^51^, ^52^ The Tinetti has also been used with individuals with ID,^53^ including DS,^45^ and is correlated with other measures of gait and gross motor skills in expected directions.^54^, ^55^

#### 2.3.3 Cognition

The Down Syndrome Mental Status Examination (DSMSE)^56^ is a measure of overall mental status and is used to assess AD‐related cognitive decline.^56^ The measure assesses recall of personal information, orientation to time, immediate and delayed memory, language, visuospatial functioning, and motor planning in individuals with DS.^3^, ^57^ The DSMSE can differentiate cognitively stable individuals with DS from those with MCI and dementia^47^ and predicts transition to dementia.^3^

#### 2.3.4 Memory

The modified Cued Recall Test (mCRT)^58^ evaluates verbal learning and memory by having participants learn and then recall a series of 12 items shown as pictures. The free recall score is the number of items the participant recalls across three trials. If an item is not freely recalled, a category cue is provided. The cued recall score is the number of items remembered after being given the category prompt across the three trials. Current analyses used the Total mCRT score, which is the sum of the free and cued recall scores. The mCRT has been shown to have high sensitivity and specificity for distinguishing between cognitively stable adults with DS and those with AD.^59^

#### 2.3.5 Dementia symptoms

The National Task Group–Early Detection Screen for Dementia (NTG)^60^ is an informant‐report of changes in function that could indicate dementia. The NTG includes 51 items across six domains: (1) activities of daily living (7 items), (2) language and communication (6 items), (3) sleep–wake change patterns (8 items), (4) ambulation (4 items), (5) memory (9 items), and (6) behavior and affect (17 items). Each item is rated on a 4‐point scale of (1) always been the case, (2) always but worse, (3) new symptom in the past year, and (4) does not apply. The purpose of the NTG is to screen for early AD clinical onset and was found to be sensitive to MCI and dementia in individuals with DS.^61^

#### 2.3.6 MRI acquisition and processing

Across sites, MRI scans were acquired on a 3T GE Discovery MR750, Siemens Trio, Siemens Prisma, or GE Signa PET/MR. High‐resolution T1‐weighted (T1w) images were collected using a 3D fast spoiled gradient echo (FSPGR) or magnetization prepared rapid acquisition gradient echo (MPRAGE) sequence (for details see Handen et al.^46^). The current study included the T1w scans that were parcellated into native space versions of the Desikan–Killiany atlas^62^ using FreeSurfer v5.3.0. Templates were formed from 12 high‐quality parcellations and then warped into each participant's native MR space using the Advanced Neuroimaging Tools (ANTs) software package.^63^, ^64^ A final native space atlas was created for each scan by determining the maximum overlap of each parceled region from the 12 warped templates. Results were visually inspected to ensure the final atlas adhered to each participant's MR anatomy. In a handful of cases, the acceptable parcellations were not produced, and thus direct application of FreeSurfer on the scan was used instead. The participant‐specific atlas was used to construct the Braak regions.^10^, ^65^ Hippocampal volume (in mm^3^) was parsed into left and right volumes and then summed for total hippocampal volume.

#### 2.3.7 PET acquisition and processing

PET scans were acquired on a PET, PET/computed tomography (CT), or PET/MRI platform certified for multicenter studies (see Handen et al.^46^), with Aβ quantified using [C‐11]Pittsburgh compound B (PiB; *n* = 101) or [F‐18]florbetapir (*n* = 42) at 50 to 70 minutes post‐injection. The tau PET scans were acquired using [F‐18]AV‐1451 (e.g., [F‐18]flortaucipir; *n* = 97) at 80 to 100 minutes post‐injection. Images were acquired in 5 minute frames, corrected for motion on a frame‐by‐frame basis using SPM8, and time averaged. Analyses focused on global Aβ. PET images were registered with their corresponding anatomical MR images. The MR scan then underwent deformable registration to the 152‐subject Montreal Neurological Institute (MNI152) template. Co‐registered PET images were warped into the MNI152 template space using the resulting transformation matrix. Standardized uptake value ratios (SUVR) were calculated to capture global amyloid burden using the standard global region, using the whole cerebellum for reference, and converted to Centiloids (see Klunk et al.^66^). The [F‐18]AV‐1451 tau images were used to calculate NFT burden; PET images were similarly co‐registered with corresponding structural T1w MRIs. Concentration of [F‐18]AV‐1451 was expressed as SUVR in the parcellation‐defined Braak regions, using cerebellar gray matter as reference.

#### 2.3.8 Clinical AD status

Clinical AD status was determined based on a case consensus conference involving a psychologist, a physician, and at least two other research staff with expertise in DSAD. Reviewers had access to all available cognitive scores, informant‐reported measure scores, medical history, recent life events, and premorbid level of ID, but were blinded to neuroimaging results and *APOE* status (see Handen et al.^46^). Participants were classified as: (1) cognitively stable, in which there was no evidence of cognitive decline beyond normal signs of aging; (2) MCI, indicating subtle and/or limited decline in cognition and/or adaptive behaviors; (3) AD, indicating significant declines in cognition or adaptive behavior over an extended period of time; or (4) unable to determine, in which changes in cognition or adaptive behaviors were not clear in consideration of significant life events or changes in medical history (as reported by the study partner).

### 2.4 Statistical analyses

Statistical analyses were completed in IBM SPSS version 28.0.1.0 and R Studio version 2023.09.1. Histograms and descriptive statistics were used to examine the distribution of variables and identify any outliers. Baseline and 32‐month Tinetti scores had a negative skew (kurtosis = 5.36). Thus, regression models were run with both raw Tinetti scores and with square root transformation scores. Findings did not differ using the transformed scores, and thus, models using raw Tinetti scores are presented to aid in interpretability. Additionally, three participants were deemed to have Tinetti change scores indicative of being outliers (> 1.5 times the interquartile range). Models were run both with and without these three participants. The overall pattern of findings did not differ when these participants were included versus excluded. Bivariate Pearson correlations, independent sample *t* tests, and one‐way analysis of variance were conducted to determine whether demographic (i.e., age, sex, race, ethnicity, trisomy type, and ID) or health history (i.e., Parkinson's disease, tremors, seizure, myoclonus, traumatic brain injury, cataracts, vision, hearing, osteoarthritis, osteoporosis, and gout) variables were associated with Tinetti scores and thus should be included as covariates in the models.

**FIGURE 1:**
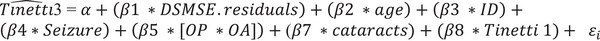
Example population model.

A series of residual change regression models was conducted to address the study aims. In all models, the 32‐month Tinetti Gait score was the dependent variable, and the baseline Tinetti score and relevant sociodemographic variables (i.e., those significantly associated with the Tinetti Gait score) were predictors. To address the first aim of the study, baseline Aβ PET, NFT PET, and MRI hippocampal volume were included as predictor variables in models. Total intracranial volume (ICV) was also included in the hippocampal volume model to control total brain size. To address aim 2, residual change in the mCRT, DSMSE, and NTG from baseline to the 32‐month follow‐up were included as predictors in models. These change scores were created by regressing out the effect of baseline mCRT, DSMSE, or NTG score from the 32‐month score. To address aim 3, the 32‐month clinical diagnoses (e.g., AD statuses) were included as predictors. Significance was set at *p* < 0.05. See Figure 1 for an example population model.

## 3 RESULTS

### 3.1 Preliminary analyses

Of the 358 adults with DS enrolled in ABC‐DS at the baseline, 218 (61%) participants completed the Tinetti Gait assessment at baseline and the 32‐month follow‐up. Independent *t* tests and chi‐squared tests indicated that the 218 participants included in analyses did not differ from those who did not complete the 32‐month follow‐up (*n* = 140) in age, sex, race, ethnicity, or ID level (*p* < 0.05; see Table S1 in supporting information). On average, participants were 44.4 years old (standard deviation [SD] = 9.20), 56% were male, and 98% self‐identified as White. Most participants had a premorbid ID level in the mild range (56%), while 35% had moderate ID and 9% had severe/profound ID. At baseline, 171 (78%) of participants were cognitively stable, 36 (17%) had MCI, 8 (4%) had dementia, and 3 (1%) had a status of unable to determine. By 32 months, 149 (68%) of participants were cognitively stable, 24 (11%) had MCI, and 41 (19%) had dementia. Three participants deemed to have MCI at baseline reverted to cognitively stable at 32 months, while all other participants with MCI at baseline (*n* = 33) either remained MCI or transitioned to dementia at 32 months. Participants classified as “unable to determine” at 32 months (*N* = 5; 2%) were excluded from the model. See Table 1 for additional demographic information.

**TABLE 1:** Participant demographic characteristics.

Age (years), M ± SD	44.4 ± 9.2
BMI, M ± SD	31.5 ± 7.2
Sex, *N* (%)	
Male	122 (56.0%)
Female	96 (44.0%)
Race, *N* (%)	
White	213 (97.7%)
Black or African American	3 (1.4%)
Asian	2 (0.9%)
Ethnicity, *N* (%)	
Hispanic or Latino	10 (4.6%)
Intellectual disability level, *N* (%)	
Mild	121 (55.5%)
Moderate	77 (35.3%)
Severe/profound	20 (9.2%)
Clinical AD status at 32 months, *N* (%)	
Cognitively stable	149 (68.3%)
MCI	24 (11%)
Dementia	41 (18.8%)
Unable to determine	5 (2.3%)
*APOE* ε4, *N* (%)	
Presence of at least one allele	50 (23%)
Trisomy type, *N* (%)	
Full	196 (89.9%)
Mosaic	10 (4.6%)
Translocation	12 (5.5%)
Orthopedic condition, *N* (%)	
Combined	67 (30.7%)
Osteoarthritis	36 (16.5%)
Osteoporosis	31 (14.2%)
Cataracts, *N* (%)	72 (33.0%)
Seizure history, *N* (%)	14 (6.4%)
Tinetti gait, M ± SD	
Baseline	11.04 ± 1.51
32‐month	10.52 ± 2.16
DSMSE, M ± SD	
Baseline	58.29 ± 16.77
32‐month	56.60 ± 19.01
mCRT, M ± SD	
Baseline	27.19 ± 10.71
32‐month	26.81 ± 10.78
NTG, M ± SD	
Baseline	6.11 ± 9.07
32‐month follow‐up	5.33 ± 8.26

Abbreviations: AD, Alzheimer's disease; *APOE*, apolipoprotein E; DSMSE, Down Syndrome Mental Status Examination; MCI, mild cognitive impairment; mCRT, modified Cued Recall Test; NTG, National Task Group–Early Detection Screen for Dementia; SD, standard deviation.

Of the 218 participants with both Tinetti Gait scores, 206 completed the DSMSE, 186 completed the mCRT, and 204 completed the NTG. Of the 218 participants, PET Aβ was available for 143 participants, PET NFT was available for 97 participants, and MRI hippocampal data were available for 81 participants. The remaining participants either did not undergo imaging or imaging was acquired but using procedures that were not harmonized with the ones used in the current analyses. Independent *t* tests and chi‐squared tests indicated that the 143 participants with imaging data did not significantly differ from those without imaging data (*n* = 75) in sex, race, ethnicity, or ID level (*p* < 0.05), but did significantly differ in terms of age (*p *< 0.05; see Table 2). The mean age of those with imaging data with significantly less ($\bar{x}$ = 42.6) than the mean age of those without imaging data ($\bar{x}$ = 48.6).

**FIGURE 2:**
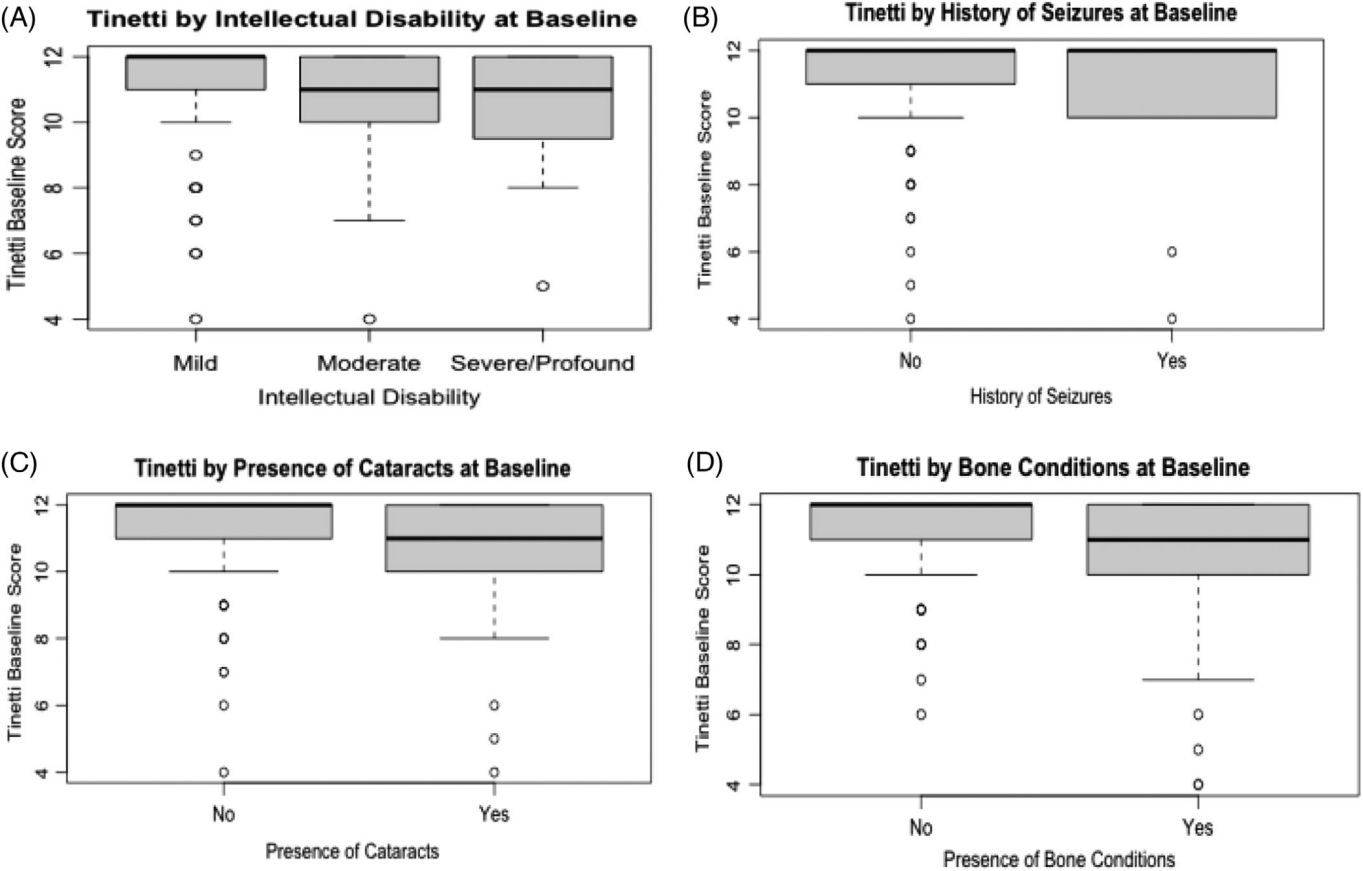
Baseline Tinetti Gait score by intellectual disability level (A), presence of seizures (B), presence of cataracts (C), and presence of orthopedic condition (D)

Table 1 displays the mean and SD for the Tinetti Gait score at baseline and the 32‐month follow‐up. Analyses were conducted to examine the association between demographic variables and the Tinetti Gait score at baseline. Age was significantly negatively associated with the Tinetti Gait score (*r *= −0.405, *p* < 0.01). Additionally, there was a significant difference in the Tinetti Gait score across ID levels (*F*[2, 215] = 5.87, *p* < 0.01). Tukey post hoc indicated that participants with moderate ID (*p* = 0.013, 95% confidence interval [CI] = [0.17, 1.88]) and severe/profound (*p* < 0.01, 95% CI = [0.38, 2.04]) had significantly lower baseline Tinetti Gait scores than those with mild ID. The presence of seizures (*t*[216] = 5.327, *p *< 0.01), cataracts (*t*[216] = 2.370, *p *= 0.02), and osteoarthritis and/or osteoporosis (*t*[216] = 6.541, *p *< 0.01) were also significantly negatively associated with Tinetti Gait scores. The remaining health conditions (Parkinson's disease, tremors, myoclonus, traumatic brain injury, vision, hearing, and gout) were not significantly related to Tinetti Gait scores (*p* > 0.05). See Table 1 for the proportion of participants with a history of seizures, cataracts, osteoarthritis, and osteoporosis. In subsequent residual change models, age, ID, and presence of seizures, cataracts, and osteoarthritis/osteoporosis were included as covariates. Also see Figure 2 for boxplots comparing the presence and absence of covariates to baseline Tinetti scores.

### 3.2 Cognitive decline

**FIGURE 3:**
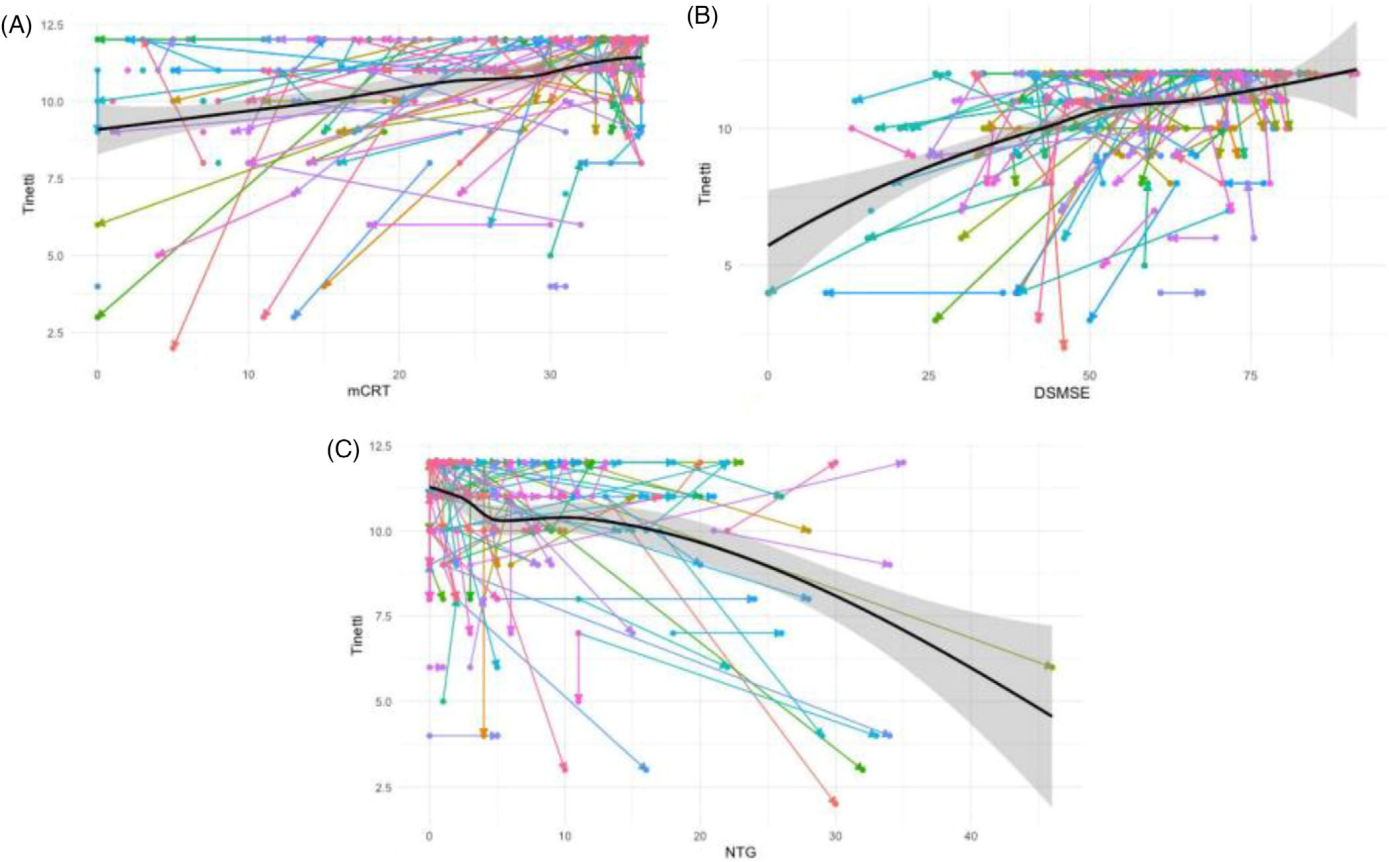
Change in cognitive functioning in relation to change in Tinetti score from baseline to 32‐month follow‐up. A, Modified Cued Recall Test (mCRT). B, Down Syndrome Mental Status Examination (DSMSE); C, National Task Group—Early Dementia Screener (NTG). Each line represents a participant. Circles denote scores at baseline and arrows denote scores at the 32‐month follow‐up. The solid black line represents the locally estimated scatterplot smoothing data trend, and the gray band represents the 95% confidence interval

Table 1 also provides the mean and SD for cognitive scores at baseline and the 32‐month follow‐up. In the residual change model, residual change in DSMSE scores significantly predicted the 32‐month Tinetti Gait score (*F*[8, 198] = 16.24, *p *< 0.01). Specifically, Tinetti Gait scores at 32 months were 0.632 points lower for every 1‐point reduction in residual DSMSE scores from baseline to the 32‐month follow‐up, with other predictors held constant (*p* < 0.01). The residual change in mCRT scores also significantly predicted Tinetti Gait scores at the 32‐month follow‐up (*F*[8, 178[ = 13.98, *p *< 0.01). Tinetti Gait scores were 0.689 points lower for every 1‐point reduction in residual mCRT scores from baseline to the 32‐month follow‐up while holding all other predictors constant (*p* < 0.01). Finally, the residual change in NTG scores significantly predicted Tinetti Gait scores at the 32‐month follow‐up (*F*[8, 196] = 17.96, *p *< 0.01). Tinetti Gait scores at 32 months decreased by 0.814 for every 1‐point increase in residual NTG reported symptoms from baseline to the 32‐month follow‐up while holding all other predictors constant (*p* < 0.01). See Table 2 for an overview of each assessment's residual change model and Figure 3 for the change in Tinetti compared to the change in each assessment.

**TABLE 2:** Regression models of change in cognitive functioning predicting gait at 32 months.

Variable	β	SE	*t* value	*p* value
DSMSE	0.632	0.139	4.817	2.89 e‐06***
Age	−0.0342	0.0158	−2.162	0.0318*
ID moderate	0.130	0.257	0.504	0.615
ID severe/profound	−0.624	0.435	−1.432	0.154
Seizure	−0.316	0.501	−0.631	0.529
Orthopedic condition	−0.565	0.308	−1.834	0.0682
Cataracts	0.0240	0.270	0.089	0.929
Tinetti baseline	0.533	0.0888	5.999	9.29 e‐09***
mCRT	0.689	0.128	5.37	2.50 e‐07***
Age	−0.0132	0.0164	−0.804	0.423
ID moderate	0.204	0.262	0.776	0.439
ID severe/profound	−0.590	0.443	−1.33	0.185
Seizure	−0.355	0.539	−0.659	0.511
Orthopedic condition	−0.737	0.313	−2.35	0.0199*
Cataracts	0.202	0.271	0.748	0.456
Tinetti baseline	0.507	0.0910	5.572	9.18 e‐08***
NTG	−0.814	0.132	−6.15	4.39 e‐09***
Age	−0.0325	0.0153	−2.12	0.0353*
ID moderate	0.140	0.248	0.565	0.573
ID severe/profound	−0.468	0.429	−1.09	0.277
Seizure	−0.0972	0.499	−0.195	0.850
Orthopedic condition	−0.573	0.299	−1.91	0.0571
Cataracts	0.639	0.263	0.243	0.808
Tinetti baseline	0.528	0.0880	6.00	9.29 e‐09***

*Note*: Orthopedic condition comprises osteoarthritis and osteoporosis.

Abbreviations: DSMSE, Down Syndrome Mental Status Examination; ID, intellectual disability; mCRT, modified Cued Recall Test; NTG, National Task Group–Early Detection Screen for Dementia; SE, standard error.

* *p* < 0.05.

*** *p*< 0.001.

### 3.3 Neuroimaging AD biomarkers

**FIGURE 4:**
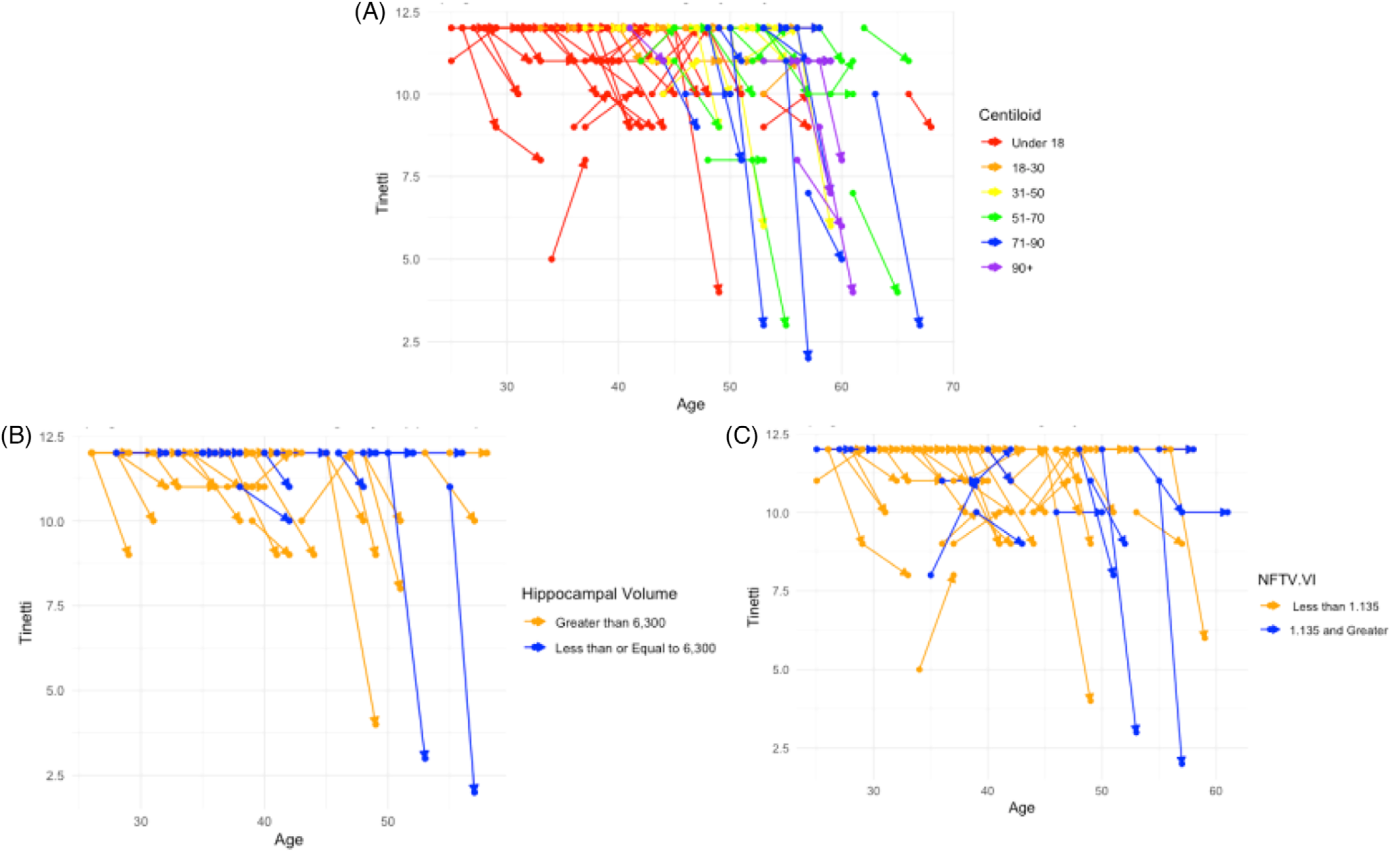
Change in Tinetti score from baseline to the 32‐month follow‐up. Each line represents a participant based on (A) amyloid burden in Centiloids, (B) low versus high hippocampal volume, (C) high versus low neurofibrillary tangles (NFTs) in Braak V and VI regions. Circles denote scores at baseline and arrows denote scores at 32‐month follow‐up

Baseline A β PET, NFT PET, and ICV controlled hippocampal volume were implemented into respective residual change models. Baseline A β significantly predicted the 32‐month Tinetti Gait score (*F*[8, 135] = 9.134, *p *< 0.01). The 32‐month Tinetti Gait scores decreased by 0.0143 points with each 1 Centiloid higher baseline A β PET, holding all other predictors constant (*p* < 0.01). Baseline tau PET was also a significant predictor of Tinetti Gait scores at 32 months. The model predicted a 3.94‐point decrease in Tinetti Gait scores for every 1 SUVR increase in baseline tau PET representing regions consistent with Braak stages I and II while holding all other predictors constant (*p* < 0.01). Additionally, Tinetti Gait scores decreased by 5.10 and 5.12 points for every 1 SUVR increase in baseline tau PET in regions representing Braak stages III and IV and V and VI, respectively, while holding all other predictors constant (*p* < 0.01). Finally, the residual change model of total hippocampal volume was found to be a significant predictor of 32‐month Tinetti Gait scores (*F*[9, 72] = 4.372, *p *< 0.01). ICV was included in this model to control total brain size. Tinetti Gait scores decreased by 9.26 e–04 points for every 1 mm^3^ reduction in hippocampal volume while holding all other predictors constant (*p* < 0.01). See Table 3 for an overview of the residual change models for A β, tau, and hippocampal volume and Figure 4 for the change in Tinetti by each neuroimaging procedure.

**TABLE 3:** Regression models of baseline AD biomarkers predicting gait at 32 months.

Variable	β	SE	*t* value	*p* value
A β	−0.0143	0.00604	−2.37	0.00288**
Age	−0.0377	0.0243	−1.55	0.0193*
ID moderate	−0.0354	0.340	−0.104	0.917
ID severe/profound	−0.686	0.507	−1.35	0.179
Seizure	−0.0365	0.762	−0.048	0.962
Orthopedic condition	−0.477	0.421	−1.13	0.259
Cataracts	0.0631	0.360	0.175	0.861
Tinetti baseline	0.571	0.149	3.83	1.97 e‐04***
NFT I–II	−3.94	1.155	−3.41	9.77 e‐04***
Age	−0.0271	0.0304	−0.891	0.375
ID moderate	0.0235	0.434	0.0540	0.957
ID severe/profound	−0.200	0.531	−0.376	0.708
Seizure	−0.0336	1.719	−0.0200	0.984
Orthopedic condition	−0.563	0.557	−1.011	0.315
Cataracts	−0.203	0.434	−0.468	0.641
Tinetti baseline	0.360	0.176	2.047	0.0436*
NFT III–IV	−5.10	1.21	−4.23	5.63 e‐05***
Age	−0.0244	0.0283	−0.862	0.391
ID moderate	−0.0476	0.416	−0.115	0.909
ID severe/profound	−0.266	0.515	−0.516	0.607
Seizure	−0.00538	1.67	−0.00300	0.997
Orthopedic condition	−0.600	0.537	−1.12	0.268
Cataracts	−0.152	0.420	−0.362	0.718
Tinetti baseline	0.332	0.171	1.95	0.0548
NFT V–VI	−5.124	1.16	−4.40	3.00 e‐05***
Age	−0.0428	0.0261	−1.64	0.105
ID moderate	−0.207	0.410	−0.505	0.615
ID severe/profound	−0.232	0.512	−0.454	0.651
Seizure	0.0861	1.65	0.052	0.959
Orthopedic condition	−0.461	0.528	−0.874	0.385
Cataracts	−0.268	0.419	−0.641	0.523
Tinetti baseline	0.340	0.169	2.00	0.0481*
Hippocampal volume	9.26 e‐04	2.36 e‐04	3.93	0.000195***
Age	−0.0303	0.0327	−0.925	0.358
ID moderate	−0.0388	0.460	−0.844	0.401
ID severe/profound	0.0201	0.613	0.0330	0.974
Seizure	1.33	1.72	0.773	0.442
Orthopedic condition	−0.0899	0.593	−0.152	0.880
Cataracts	−0.110	0.500	−0.220	0.826
Intracranial volume	−4.25 e‐06	1.59 e‐06	−2.67	0.00942**
Tinetti baseline	0.640	0.318	2.01	0.0484*

*Note*: Orthopedic condition comprises osteoarthritis and osteoporosis.

Abbreviations: A β,a myloid beta; AD, Alzheimer's disease; ID, intellectual disability; NFT, neurofibrillary tangle; SE, standard error.

* *p* < 0.05.

** *p* < 0.01.

*** *p*< 0.001.

### 3.4 Clinical AD status

**FIGURE 5:**
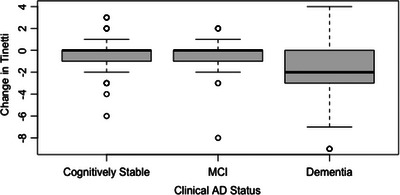
Change in Tinetti scores from baseline to the 32‐month follow‐up by clinical Alzheimer's disease (AD) status at the 32‐month follow‐up. The *y* axis represents the change in Tinetti scores from baseline to the 32‐month follow‐up. Negative numbers represent a decrease Tinetti scores and positive numbers represent an increase in Tinetti scores

In residual change models, clinical AD status significantly predicted the 32‐month Tinetti Gait score (*F*[10, 197] = 17.22, *p *< 0.01). Compared to participants who remained cognitively stable, participants who had dementia scored an average of 2.12 less points on the Tinetti Gait assessment (*p *< 0.01). However, Tinetti scores for those with MCI were not significantly different from participants who remained cognitively stable (*p *= 0.34). See Table 4 for an overview of the clinical AD status residual change model, and Figure 5 for boxplots of clinical AD status by change in Tinetti.

**TABLE 4:** Regression model of change in clinical AD status predicting gait at 32 months.

Variable	β	SE	*t* value	*p* value
MCI	−0.377	0.398	−0.948	0.344
Dementia	−2.12	0.364	−5.81	2.51 e‐08***
Age	−6.04 e‐03	0.0173	−0.350	0.727
ID moderate	0.204	0.255	0.799	0.425
ID severe/profound	−0.382	0.424	−0.903	0.368
Seizure	−0.0820	0.305	−0.168	0.869
Orthopedic condition	−0.794	0.305	−2.61	0.00987**
Cataracts	0.0864	0.266	0.325	0.745
Tinetti baseline	0.529	0.0862	6.135	4.73 e‐09***

*Note*: Orthopedic condition comprises osteoarthritis and osteoporosis.

Abbreviations: AD, Alzheimer's disease; ID, intellectual disability; MCI, mild cognitive impairment; SE, standard error.

** *p* < 0.01.

*** *p*< 0.001.

## 4 DISCUSSION

Gait impairments are a prominent feature of AD, often beginning prior to dementia onset in the general adult population.^21^, ^22^ The present study was the first longitudinal study to assess change in gait in relation to neuroimaging biomarkers of AD pathologies, cognitive decline, and AD clinical status in adults with DS. Overall, findings suggest that gait impairments have robust associations with AD pathology and symptomology in DS and thus are part of the broader array of functional declines evident in DSAD that should be included in DSAD screening efforts.

Adults with DS who had higher Aβ and NFT burden at baseline evidenced more gait impairments at 32 months than those with lower Aβ and NFT burden. These results are in line with findings on late‐onset sporadic AD (LOAD) and autosomal‐dominant AD, in which higher A β and NFT burden is associated with slower gait speed,^34^ decreased cadence,^67^ and lower overall gait quality.^68^, ^69^ In the current study, NFT in Braak stages III and IV and V and VI had the greatest impact on Tinetti Gait scores, suggesting that NFT accumulation in limbic and neocortical areas may take a toll on gait. Hippocampal atrophy was also associated with gait impairments across time in adults with DS, mirroring findings in the general population of older adults.^70^

The current study also examined associations between gait performance and AD‐related cognitive declines in DS. Even after controlling for age, premorbid ID level, and medical conditions that can impact gait (seizures, orthopedic conditions, cataracts), greater decline in gait performance across the 32 months was associated with cognitive declines (as indicated by the mCRT and DSMSE) and increases in informant‐reported dementia symptoms (NTG). In addition, adults with DS who were deemed to have dementia at 32 months (15% developed dementia during the study period while 8% had dementia at baseline) had lower Tinetti Gait scores than those who were cognitively stable. However, Tinetti Gait scores for participants with MCI at 32 months (7% developed MCI during the study period while 3% had MCI at baseline) were not significantly different from those who were cognitively stable. Impairments in gait were most strongly associated with later stages of tau PET NFT burden (i.e., spread across the neocortex). Thus, gait impairments may be observed closer to the onset of dementia than MCI in adults with DS, at least using observable gross measures of gait. However, it is also possible that the limited number of adults with DS and MCI (*N* = 24) obscured detection of more subtle impairments. Instead, more precise gait measures (e.g., technology‐based gait mats) may be more sensitive to declines in gait that precede or correspond with onset of MCI in DS. Overall, these findings are consistent with research on LOAD outside of DS in which gait impairments are evident prior to dementia early during the unfolding of AD symptomology, with increasing severity from early to late stages of dementia.^71^, ^72^, ^73^, ^74^, ^75^

The current study had several strengths. Analyses leveraged a large natural history cohort of adults with DS, and the cognitive and neuroimaging protocols were harmonized across data collection sites. The clinical AD status groups were based on a consensus process that drew on a large battery of direct and informant‐reported assessments. Additionally, this study provides robust and multimodal evidence that declines in gait are linked to AD pathology. There were also limitations to the current study. First, the gait assessment was based on observational coding. Raters complete a comprehensive neuropsychological and gait training, but no inter‐rater reliability has been established across sites. Second, only some of the ABC‐DS data collection sites included the neuroimaging biomarkers of interest, which reduced the sample size for some models. A small number of attempted MRI scans were uninterpretable due to participant movement, but it is possible that this altered sample characteristics to be more representative of higher functioning adults with DS. It should be noted that the ABC‐DS protocol includes data collection on participants every 16 months. However, neuroimaging biomarkers (Aβ, NFT, and hippocampal volume) are only obtained at baseline and 32‐month visits. Thus, current analyses focused on change across 32 months (baseline to month 32 visit) given our inclusion of neuroimaging biomarkers. In addition, this longer duration (32 vs. 16 months) was deemed to be better suited for detecting changes in cognition and gait. That said, future studies that include longer time intervals and additional data points are needed to better capture individual change trajectories.^76^ Neuroimaging data were only available on a subset of the sample and this subset was younger than those without imaging data. Thus, neuroimaging findings may not fully capture effects in later disease stages. Finally, findings are not representative of non‐verbal adults with DS and/or those with a mental age of < 3 years given study inclusion criteria. Future studies should include adults with DS with a broader range of racial/ethnic diversity backgrounds, which is a goal of the ABC‐DS study's current recruitment efforts. Future studies should also focus on regional amyloid, tau, and neurodegeneration outcomes to better understand mechanisms underlying the associations between gait change and clinical and neuropathological progression of AD in people with DS. Additionally, future studies using more sensitive technology‐based gait mats may be able to detect even earlier subtle changes in gait related to AD pathology and symptomology. Finally, it will also be important for future studies to examine which specific domains of gait (e.g., cadence, foot clearance, and step symmetry) are impacted by AD pathology and are best able to distinguish among DSAD clinical status groups.

In summary, our findings suggest that gait impairments are a key feature of DSAD. The onset of gait impairments is associated with elevated Aβ and NFT burden and hippocampal atrophy and corresponds with early cognitive declines, dementia symptoms, and clinical AD status. In particular, 1 SUVR tau PET change in brain regions corresponding to NFT III and IV and NFT V and VI was associated with an ≈ 40% decrease in Tinetti scores, demonstrating Tinetti's sensitivity to NFT burden. These findings may also have important clinical implications, as gait assessments could serve as a screening tool for AD detection in adults with DS. The Tinetti Gait Test may offer a quick, cost‐effective, non‐invasive screen for gait impairments that occur as part of the evolution of AD symptomology in adults with DS. Additionally, caregivers may consider monitoring changes in gait, potentially lending early insight to cognitive decline and fall risk. Future studies are needed to further establish the relationship between gait and AD development in adults with DS.

## CONFLICT OF INTEREST STATEMENT

M.M. received royalties from the University of Rochester and consulting fees from NovoGlia, Inc. and Ireno Health, PBC. S.Z. received royalties from Pavilion Publishing for CAMDEX‐DS‐II, paid to the Horizon‐21 Research Consortium. S.H. received consulting fees from Ionis Pharmaceuticals. M.Z. received consulting fees from LuMind IDSC. E.H. received consulting fees from Cyclo Therapeutics, Alzheon, and Elsevier.

## CONSENT STATEMENT

Informed consent and/or assent were obtained for all participants.

## Supporting information



Supporting Information

Supporting Information

Supporting Information
