# Supplementary material for: Longitudinal investigation of gait and Alzheimer's disease in adults with Down syndrome
Source: Alzheimers Dement. 2025 Apr 28;21(4):e70211. doi: 10.1002/alz.70211 (PMC12035545; doi:10.1002/alz.70211)
Supplement: Supplementary file 2 — Supporting Information [file ALZ-21-e70211-s002.docx]

Supplement Table 2. Difference in demographics between those with and without neuroimaging data.

| **Variable** | **Test** | **Result** |
| --- | --- | --- |
| Age | Independent t-test | t = - 4.668 * |
| Sex | Chi-squared | X^2^ = 2.571 |
| Race | Chi-squared | X^2^ = 0.444 |
| Ethnicity | Chi-squared | X^2^ = 0 |
| Intellectual disability | Chi-squared | X^2^ = 5.832 |

Significance (p<0.05) is indicated by *
